# Evaluating biosecurity implementation in commercial broiler poultry production in Gujarat

**DOI:** 10.3389/fvets.2025.1699509

**Published:** 2026-01-28

**Authors:** Bhoomika Joshi, Khushbu Rana, Akash Golaviya, Subhash Jakhesara, Prakash Koringa, Ayona Silva-Fletcher, Fiona Tomley, Haidaruliman Paleja

**Affiliations:** 1 Department of Veterinary Biotechnology, College of Veterinary Science and Animal Husbandry, Kamdhenu University, Anand, Gujarat, India; 2 Department of Veterinary Biotechnology, College of Veterinary Science and Animal Husbandry, Kamdhenu University, Himmatnagar, Gujarat, India; 3 Royal Veterinary College, University of London, London, United Kingdom

**Keywords:** biosecurity, broiler poultry, Gujarat, antimicrobial resistance, commercial poultry farming

## Abstract

**Introduction:**

Poultry farming is a critical livelihood and nutrition source in India, particularly with the rapid expansion of commercial broiler production. As operations grow, strong biosecurity practices are essential to protect flock health, reduce antibiotic use, and prevent disease outbreaks. However, limited information exists on how effectively biosecurity measures are implemented in many developing regions. This study evaluated the current status of biosecurity practices in commercial broiler farms in Gujarat, India.

**Methods:**

A structured questionnaire was administered to collect data on farmer demographics, farm characteristics, and biosecurity practices. Descriptive statistics, graphical summaries, and multivariate analyses were used to assess overall trends, identify weaknesses, and highlight key challenges in biosecurity implementation.

**Results:**

Most surveyed farms were medium-scale operations, though notable variation existed in flock size and management. Farmers were generally educated and primarily depended on poultry farming for income. Basic practices such as vaccination, manure disposal, and bird isolation were widely recognized. However, major gaps were identified in rodent control, carcass disposal (with 97% relying solely on burial), vehicle sanitation (absent in 53.85% of farms), and awareness of antimicrobial resistance (AMR), which was low among 79.49% of respondents. Over half of the farmers had never received formal biosecurity training, and many reported challenges related to limited skilled labor, financial constraints, and inadequate veterinary support.

**Discussion:**

The findings demonstrate widespread reliance on poultry farming but highlight inconsistent adoption of critical biosecurity measures. Poor awareness of AMR, limited training, and infrastructural deficits pose substantial risks for disease spread and antibiotic dependence. Targeted interventions—including capacity-building programs, improved veterinary access, and affordable biosecurity tools—are necessary to strengthen farm resilience and support public health goals through reduced antimicrobial reliance and enhanced disease prevention.

## 1 Introduction

Poultry farming refers to the agricultural sector that raises poultry for meat and eggs, which is regarded as the most important means of alleviating poverty and ensuring food and nutrition security, especially in developing countries (1). India makes up approximately 7% of global egg production, 2.24% of the world's total meat production according to the 20th Livestock Census (2). India ranks third for egg production and sixth for poultry meat production, which increased by 6.19% and 6.89% compared to the previous year in egg and poultry production, respectively. Gujarat ranks 12th in poultry birds with 21.7 million birds in the state, among which a total of 17.5 million are commercial poultry birds (3). Broiler poultry is the most popular form of chicken raised for both meat consumption and commercial purposes and is a well-known poultry farming industry with proven economic potential. Broilers have a high capacity for converting feed into food products quickly and effectively over a short period of time, roughly 1.5–2 months. As a result, they grow very quickly and have relatively high nutritional requirements due to their high rate of output (4).

Misuse and overuse of antimicrobials lead to the emergence of antimicrobial-resistant microorganisms. More than 70% of all antimicrobials marketed globally are believed to be utilized in food-producing animals (5). The poultry industry, in particular, has been related to regular use of antimicrobials. The World Health Organization's (WHO) global action plan on antimicrobial resistance (AMR) emphasizes the “One Health” approach to tackle AMR. This concept views humans, animals, the food chain, the environment, and their relationships as a single unit. Many of the same microorganisms infect both animals and people since they share the same ecosystem. Efforts from a single sector cannot prevent or eliminate the problem. Drug-resistant microorganisms can be transmitted between animals and humans through direct contact or through contaminated food; therefore, a well-coordinated approach in humans and animals is crucial to efficiently limit it (6).

Biosecurity refers to the prevention of the entrance and/or spread of dangerous organisms in an animal/bird population but also to prevent potential zoonoses (6). Experts and organizations must rethink and reinvent ways to integrate and coordinate their efforts since zoonoses develop from the dynamic confluence of humans, animals and their products, environment, agriculture, wildlife, vectors, food, water, antimicrobial usage, and changing ecosystems. These include embracing system thinking, making new investments in preventive, enhancing global infrastructures for human and animal health as well as the surveillance systems that go along with them, enhancing human capacity and skill sets, and combining communities and resources from many One Health domains. Zoonoses must be considered in our national security strategy and require corresponding expenditures in preparedness, prevention, research, and resilience due to the rising costs and social disruptions associated with outbreaks and pandemics (7). Poultry is a significant bacterial agent reservoir and disease transmission can occur through infected poultry. Pathogenic bacteria may cause infectious illness, which is the leading cause of death in both animals and humans. Because of the rising prevalence of infectious illnesses, the use of antibiotics has become the most prevalent in health care (8). Campylobacters are zoonotic bacteria that infect a broad variety of animals, particularly poultry. Undercooked poultry consumption is frequently cited as a contributory factor associated with human campylobacteriosis and is frequently given a significant role in the epidemiology of human campylobacteriosis, while other sources could additionally have a role in human illnesses (9). Salmonella spp. and *Escherichia coli* infect poultry in a variety of ways, causing digestive system disorders in both animals and humans. Addressing weaknesses and shortcomings in the poultry distribution network (PDN) and implementing robust biosecurity policies on poultry farms has been proven to be highly effective in controlling disease outbreaks, improving overall productivity, and reducing the use of antibiotics. These measures are essential for ensuring the safety and health of both animals and consumers alike. By taking proactive steps to strengthen the poultry supply chain and minimize risk factors, we can help ensure a more secure and sustainable future for this vital industry (10, 11).

The objective of the study was to assess practice of biosecurity measures on commercial broiler farms of Gujarat. It also examined the day-to-day operations of these farms, the farmers involved, and the measures they have in place to protect their flocks. Through pinpointing what works well and where gaps and pitfalls exist, our aim is to offer practical, evidence-based suggestions which may further improve on-farm biosecurity- with ultimate positive outcomes for the birds and their guardians and for the folk who ultimately rely on poultry for food.

## 2 Materials and methods

### 2.1 Study area and background

**Figure 1:**
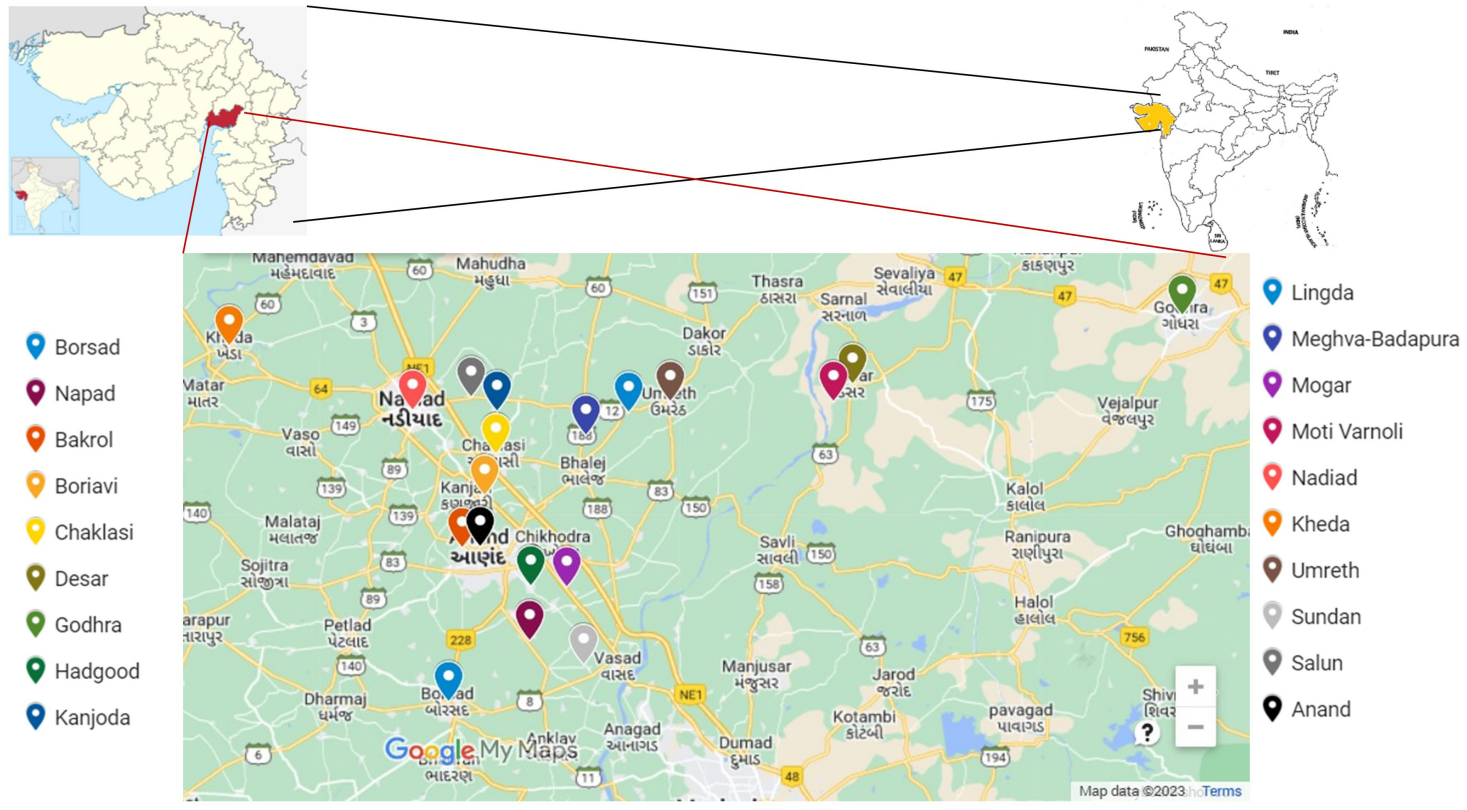
Geographic distribution of the poultry farms included in the study. The highlighted region in Gujarat, India, indicates the districts and villages from which farmers were invited for the training program and subsequently surveyed to evaluate biosecurity practices.

A one-day capacity-building training program on poultry biosecurity was conducted on 1 February 2023 at the Department of Veterinary Biotechnology, College of Veterinary Science and Animal Husbandry, Kamdhenu University, Anand, Gujarat, India. The training targeted commercial broiler farmers to enhance awareness and adoption of biosecurity measures. The study area lies at an altitude of 39 m above mean sea level, at 22°34′N latitude and 72°56′E longitude (Figure 1).

A total of 39 small- to medium-scale male broiler farmers participated in the program. The training comprised multiple expert-led sessions covering good chick selection, brooding management, common poultry diseases, vaccination, antimicrobial resistance (AMR), and farm hygiene and biosecurity. Participants were informed about the objectives of the training, and consent was obtained before data collection.

To evaluate the impact and gather information, participants were provided with a pre-training questionnaire, a biosecurity scorecard, a poultry distribution network (PDN) exercise, and a post-training questionnaire. These tools enabled data collection on baseline knowledge, farm practices, perceptions, and areas for improvement in biosecurity implementation.

### 2.2 Questionnaire design

Both questionnaires were designed in Gujarati to facilitate accurate responses and included a combination of close-ended and open-ended questions. The pre-training questionnaire captured demographic and farm-related information, while the post-training questionnaire assessed farmers' opinions, attitudes, and perceived constraints regarding biosecurity practices.

#### 2.2.1 Questionnaire 1

The first structured questionnaire focused on existing biosecurity practices adopted by commercial poultry farms, covering conceptual, structural, and operational aspects of biosecurity along with farm characteristics. It consisted of three parts:

1. Farmer profile
2. Farm profile
3. Biosecurity profile

Detailed parameters included in each part are provided in Supplementary Table S1.

#### 2.2.2 Questionnaire 2

The second questionnaire focused on farmers' perspectives and constraints in implementing biosecurity measures. It explored their understanding, attitudes, and reasons for non-adoption of specific measures, to identify practical barriers and areas requiring improvement. The topics included are listed in Table 1, and the biosecurity measures common to both questionnaires are presented in Table 2.

**Table 1:** Topics covered in the second questionnaire on farmers' perspectives toward implementing biosecurity measures.

**Sr. No**.	**Topics**
1	Major diseases/problems faced in poultry farming can be prevented by strict biosecurity
2	Possible route for transmission of diseases
3	Organs of chicken responsible for immunity
4	The biosecurity measure to be followed for poultry farming
5	Knowledge about AMR

The questionnaire assessed farmers' opinions on following biosecurity measures and explored reasons for non-implementation, providing insights for potential improvement.

**Table 2:** Biosecurity measures included in both questionnaires.

**Response**	**Biosecurity measures**
A	Location and design of poultry farm
B	Restriction on vehicle entry into the farm
C	Restriction on entry of visitor
D	Cleaning and disinfection
E	Isolation and quarantine of new birds
F	Proper manure disposal
G	Health management
H	Documentation and record-keeping
I	Good nutrition
J	Disposal of carcass
K	Action during a disease outbreak
L	Vaccination
M	Prevention of contact with wild birds
N	Rodent and pest control

This table lists the biosecurity measures assessed across both farmer questionnaires. Each measure was coded (A–N) and used to evaluate farmers' practices, perceptions, and challenges in implementing biosecurity on commercial poultry farms.

### 2.3 Poultry production and distribution network and biosecurity scorecard

The Poultry Distribution Network (PDN) exercise was used to identify key epidemiological nodes where poultry diseases are most likely to emerge and spread—namely farms, transportation routes, and markets. Participants were divided into groups of five–seven members, and each group identified and ranked critical risk points; responses were recorded by the group leader.

The biosecurity scorecard was adapted from the Poultry Disease Prevention Checklist (12) and included five sections:

1. Farm entry protocols
2. Farm security and visitor control
3. Sanitation practices
4. Manure management
5. Disease surveillance and vaccination procedures

Scores ≥80 indicated outstanding performance, 70–79 excellent, 60–69 good, 50–59 fair, and < 50 indicated a need for improvement. This tool was used to systematically evaluate and compare biosecurity performance among farms.

### 2.4 Data analysis

Responses were translated into English and entered into Microsoft Excel for data management and cleaning. Quantitative variables were analyzed using descriptive statistics (mean, standard deviation, standard error), and means were compared using one-way ANOVA. Categorical variables were expressed as frequencies and percentages, and associations between categorical variables were tested using the Chi-square test.

Factor Analysis of Mixed Data (FAMD) was performed using the *FactoMineR* package in RStudio (version 4.2.2), and results were visualized with the *Factoextra* package. Likert-scale responses were graphically represented using Microsoft Excel. Statistical significance was set at *p* < 0.05.

## 3 Results

### 3.1 Farmer profile

The farmers' profiles revealed that the majority of farmers were married (94.87%). Most families were joint families (94.9%), with only 5.1% belonging to nuclear families. A significant portion of farmers (76.92%) had less than 15 years of experience, while 17.95% had between 15 and 30 years, and 5.13% had more than 30 years of experience.

The age distribution was balanced: 44% of farmers were aged 20–35 years, 44% were aged 35–50 years, and 13% were over 50 years. All surveyed farmers had formal education. Among them, 51% completed secondary education, 13% had higher secondary, 28% were graduates, and 8% had postgraduate qualifications.

Poultry farming was the sole income source for 64% of respondents. About 33% combined poultry with agriculture, and a small fraction (2.56%) had additional jobs.

### 3.2 Farm profile

The flock size ranged from 0 to 130,000 birds, with a mean of 20,579.49 (SD = 5,064.27). Medium-scale farmers (5,000–10,000 birds) made up 46% of the sample, large-scale (>10,000 birds) 21%, and small-scale (< 5,000 birds) another 21%.

Most farms (69.2%) were operated independently, and 30.8% under contract arrangements. Funding was primarily from personal funds (74.4%), followed by bank loans (12.8%), a combination of both (7.7%), and loans from moneylenders (5.1%).

Farmers reported an average of 5.13 production cycles per year. The number of sheds ranged from 1 to 80 (mean = 5.49), and shed capacity ranged from 2,000 to 400,000 birds (mean = 31,858.97). Managerial staff averaged 1.62 per farm, while labor staff averaged 6.10.

Store rooms were present in 92.31% of farms, and 71.79% were rodent-proof. Footbaths were available in 92.31% of farms, but 53.85% lacked vehicle sanitization at entry. Proper fencing was present in 94.87% of farms. All farms had waste disposal facilities. While 79.49% had no water bodies within 500 m, 94.87% had trees around the farm, and 2.56% of farms still allowed entry of wild birds.

### 3.3 Biosecurity profile

Biosecurity practices among 39 commercial broiler farms in Gujarat varied considerably (Table 3). Farmers' knowledge was generally poor, with only 15% reporting very good to excellent understanding, and awareness of AMR was low (21%). Cleaning and disinfection measures were consistently followed, including shed sanitation, pest and rodent control, and disinfection procedures. Isolation and quarantine were partially adopted: while most farmers isolated sick birds (100%) and new birds (87%), only 41% maintained the recommended 21-day downtime between flocks. Restricted access was weakly enforced, with 31% restricting vehicle entry, 56% restricting visitors, and 59% preventing contact with wild birds. Waste and manure management showed gaps, as 97% disposed of dead birds by burial but only 21% stored manure appropriately. Health management practices were strong, with universal vaccination, veterinary advice, and protective gear use, though adoption of ethnoveterinary medicine was low (18%). A full parameter-wise breakdown is provided in Supplementary Table S2.

**Table 3:** Summary of biosecurity practices among commercial broiler farms in Gujarat.

**Biosecurity category**	**Key practices (highlights)**	**Overall adoption (%)**
Farmers' knowledge	41% very poor knowledge; 26% good; 15% very good–excellent	Varied (very poor to excellent)
Awareness of AMR	21% aware of AMR risks	21
Cleaning and disinfection	100% shed cleaning and disinfection; 72% use water sanitizer	72–100
Isolation and quarantine	87% isolate new birds; 41% keep shed empty 21 days; 100% isolate sick birds	41–100
Restricted access	31% restrict vehicles; 56% restrict visitors; 59% prevent wild bird contact	31–59
Waste and manure management	97% bury dead birds; 95% proper manure disposal; 21% store manure	21–97
Health management	100% vaccination and vet advice; 79% provide good nutrition; 18% adopt ethnoveterinary medicine	79–100

This table presents an overview of biosecurity practices grouped into major categories: farmers' knowledge, awareness of antimicrobial resistance (AMR), cleaning and disinfection, isolation and quarantine, restricted access, waste and manure management, and health management. Key adoption levels and highlighted practices are shown. A detailed parameter-wise breakdown is provided in Supplementary Table S2.

Among reported diseases, *Colisepticaemia* (92.3%), *Avian influenza* (64.1%), and *Infectious Bursal Disease* (48.7%) were most commonly observed. All farms had experienced disease outbreaks. Notably, 100% reported not using antimicrobials, and 82.05% did not adopt ethnoveterinary practices. However, all farmers sought veterinarian guidance.

### 3.4 Factorial analysis of mixed data

**Figure 2:**
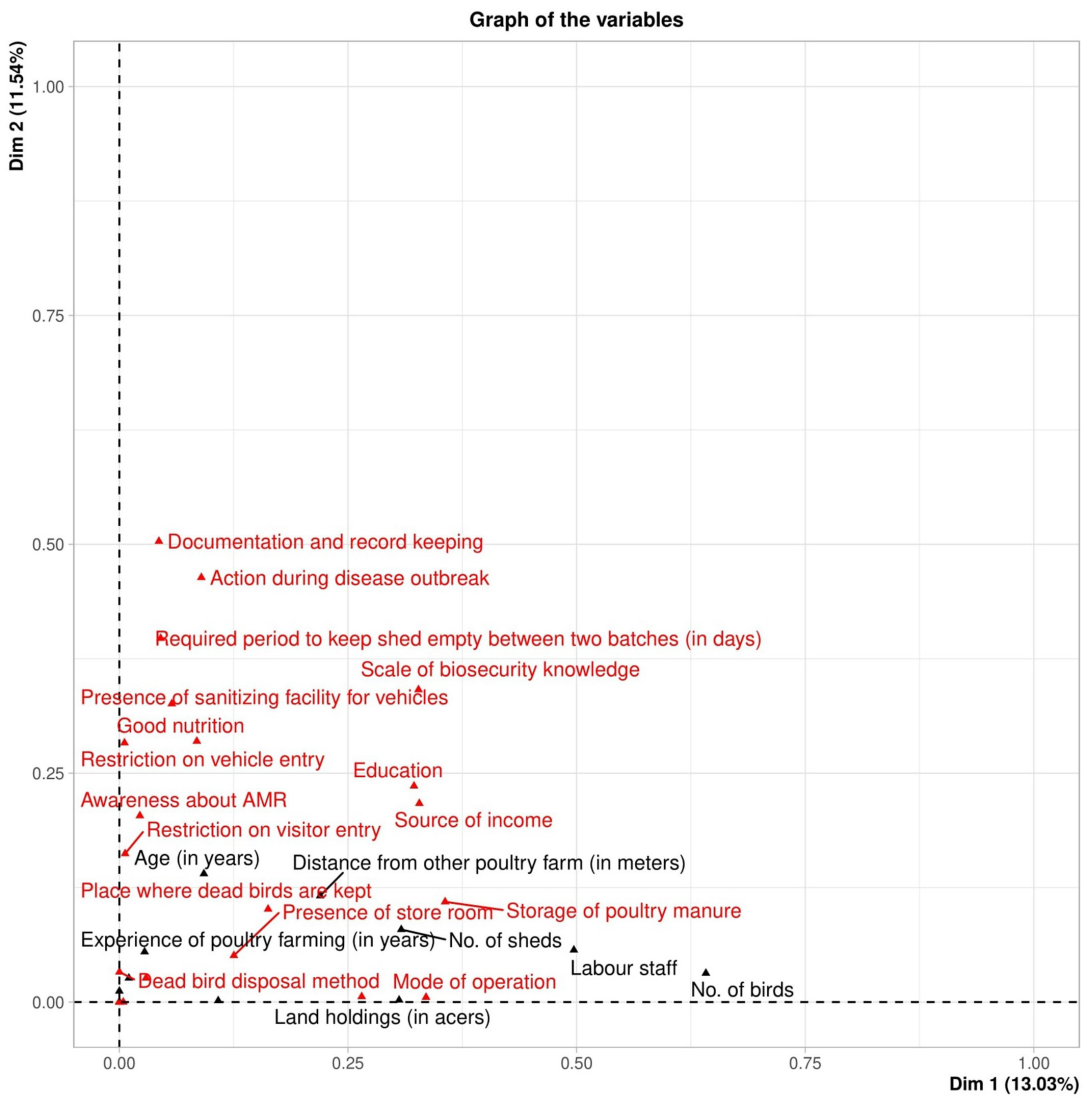
The graph of variable distribution among dimension 1 and dimension 2, the variables in black color are quantitative variables while the variable in red are qualitative variables. The graph shows that the farther away the variables are plotted on a graph from a specific dimension, the more they impact the variance for that dimension.

The scree plot in Graph 1 (Figure 2) revealed that Dimensions 1 and 2 accounted for the most variance. Dimension 1 was driven by the number of birds and labor staff; Dimension 2 was influenced by documentation, disease outbreak response, and downtime between flocks. Variables plotted farther from the axis contributed more to their respective dimensions.

### 3.5 Hierarchical cluster analysis (HCA)

**Figure 3:**
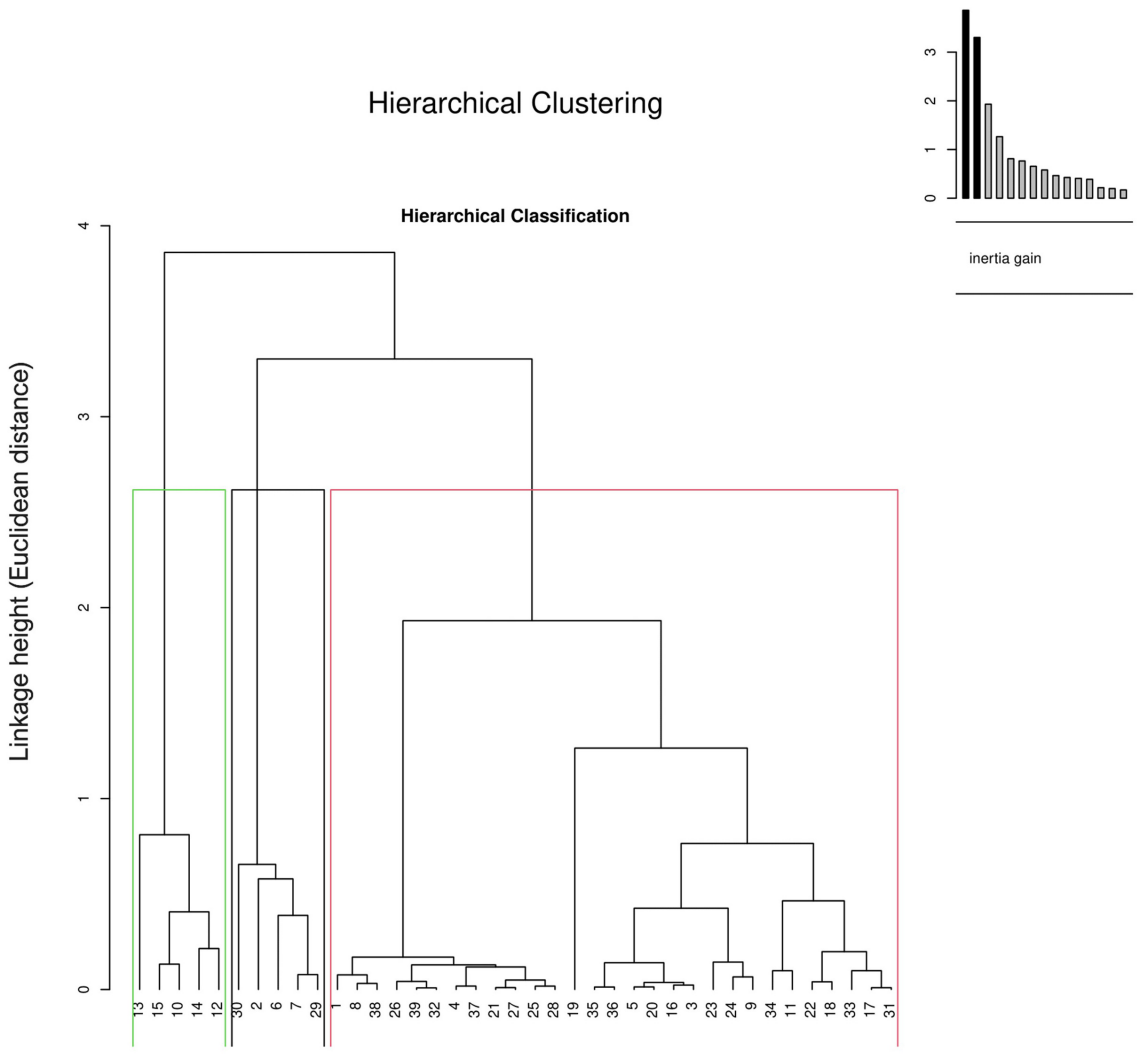
Hierarchical clustering on principal components (HCPC) of commercial broiler farms in Gujarat based on farm, farmer, and biosecurity characteristics. The dendrogram illustrates the hierarchical classification of farms into clusters with similar biosecurity profiles, while the inset barplot shows inertia gain, indicating the optimal number of clusters. The analysis was performed using the *FactoMineR* package in R (version 4.2.2).

**Figure 4:**
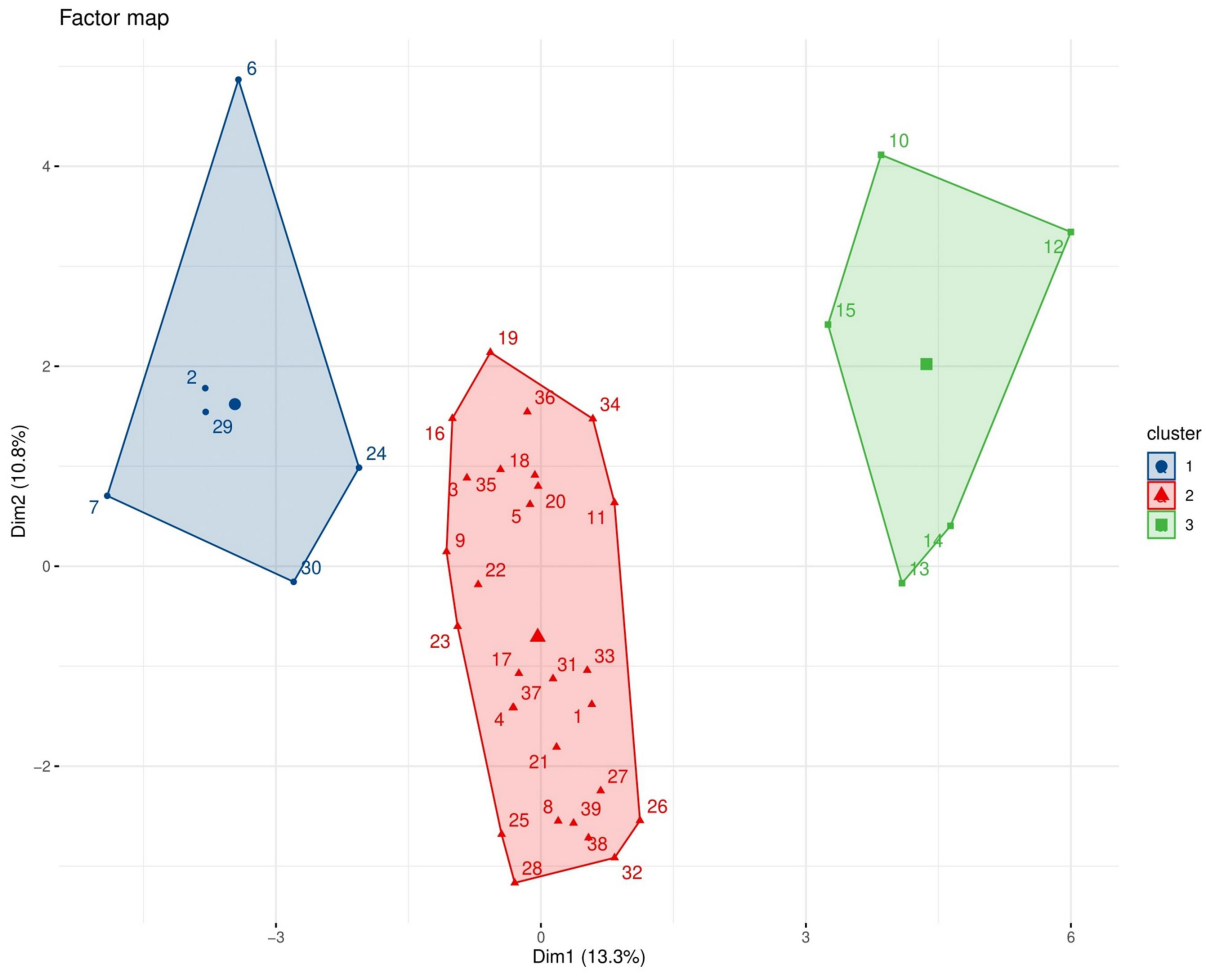
Factor map of commercial broiler farms in Gujarat obtained from hierarchical clustering on principal components (HCPC). The three clusters of farms are shown in different colors (blue: Cluster 1, red: Cluster 2, green: Cluster 3), representing distinct farm, farmer, and biosecurity profiles. The first two principal dimensions (Dim1 and Dim2) explain 13.3 and 10.8% of the variance, respectively. Cluster 2 included the majority of farms, whereas Clusters 1 and 3 were smaller, reflecting more distinct biosecurity profiles. Analysis was performed using the *FactoMineR* package in R (version 4.2.2).

Hierarchical Clustering in Graph 2 (Figure 3) shows distribution of farms in to three clusters by the Euclidean distance method among the individuals based on the characteristics. While the Graph 3 (Figure 4) displaying the cluster plot shows the placement of the three clusters in two dimensions. Cluster 1 included 74.35% of farms; Cluster 2 and 3 comprised the rest equally.

- Cluster 1: 75% stored poultry manure, none had footbaths, all respondents were postgraduates. Alarmingly, all kept dead birds inside sheds and 31.25% had very poor biosecurity knowledge.
- Cluster 2: 83.87% did not store manure, 77.78% had footbaths, and none were postgraduates.
- Cluster 3: 41.67% were in contract farming, 66.67% lacked store rooms, and none ran their own farms.

The FAMD results showed that the first five dimensions captured the primary structure of the dataset (Table 4). Dimension 1 explained 13.03% of the variance, followed by Dimension 2 with 11.54%. These two dimensions were retained for further visualization and interpretation of farm, farmer, and biosecurity characteristics.

**Table 4:** Eigenvalues and variance explained by the first five dimensions from Factor Analysis of Mixed Data (FAMD).

**Dimension**	**Eigenvalue**	**Variance (%)**
Dim.1	4.82	13.03
Dim.2	4.27	11.54
Dim.3	3.17	8.58
Dim.4	2.63	7.11
Dim.5	2.48	6.71

The table shows eigenvalues and the percentage of variance explained by the first five dimensions retained in the FAMD analysis of farm, farmer, and biosecurity characteristics. Dimensions 1 and 2 explained 13.03% and 11.54% of the variance, respectively, and were used to construct the factor maps.

### 3.6 Post-training questionnaire

**Figure 5:**
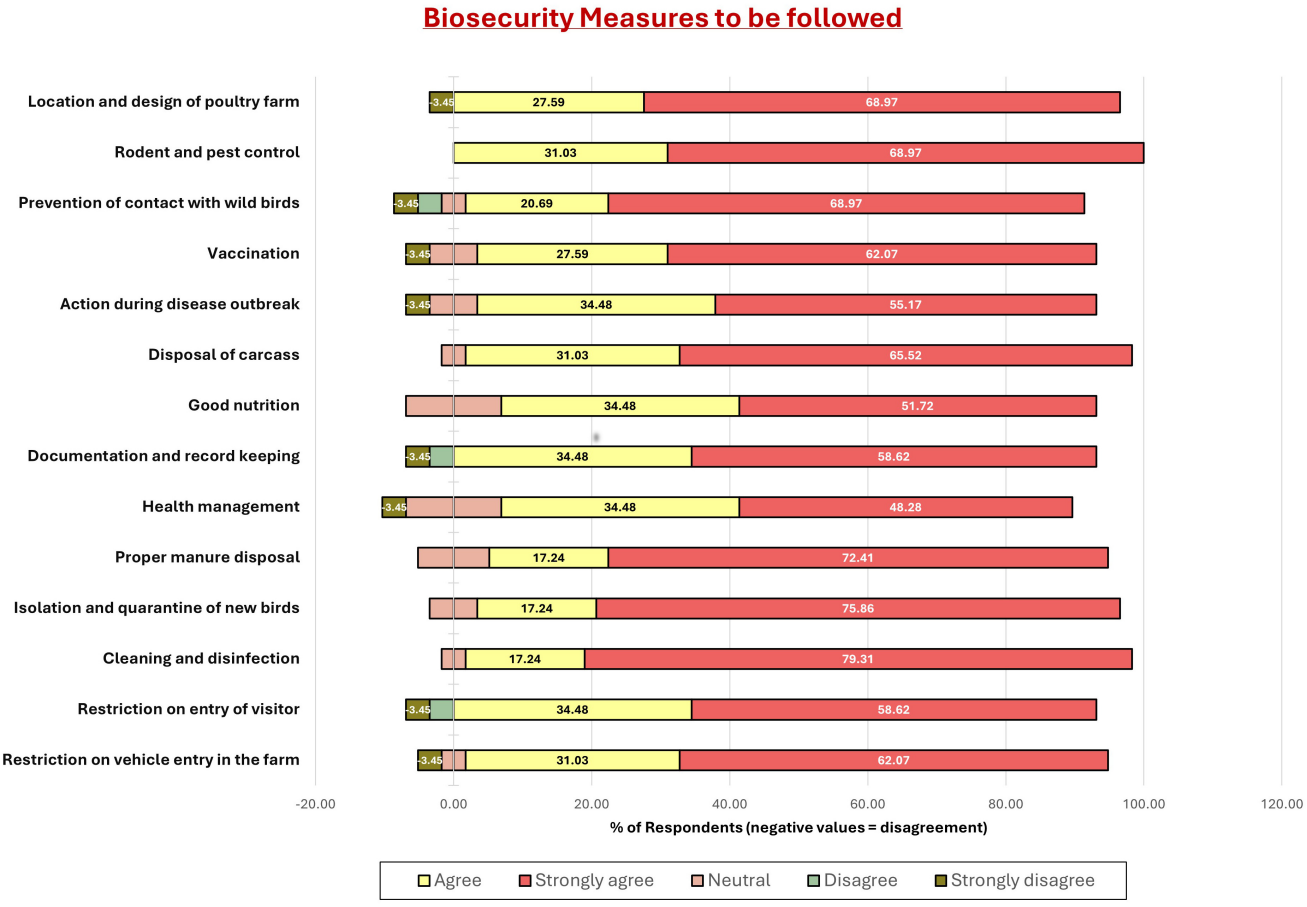
Responses of poultry farmers in Gujarat regarding recommended biosecurity measures. Bars represent the proportion of farmers who strongly agreed, agreed, were neutral, disagreed, or strongly disagreed with each statement. Most farmers strongly agreed with measures such as rodent and pest control, vaccination, carcass disposal, and proper manure management, while fewer expressed strong agreement with documentation, restriction of visitor entry, or quarantine of new birds. When compared with Figure 6, this highlights a gap between farmers' recognition of the importance of certain measures and their perceived ability to implement them.

Graph 4 (Figure 5) showed strong agreement (>70%) on the importance of manure disposal, nutrition, and health management. Isolation, quarantine, and disease response also had positive responses. Areas like vehicle entry restrictions, wild bird contact, and carcass disposal had divided views, indicating need for awareness.

**Figure 6:**
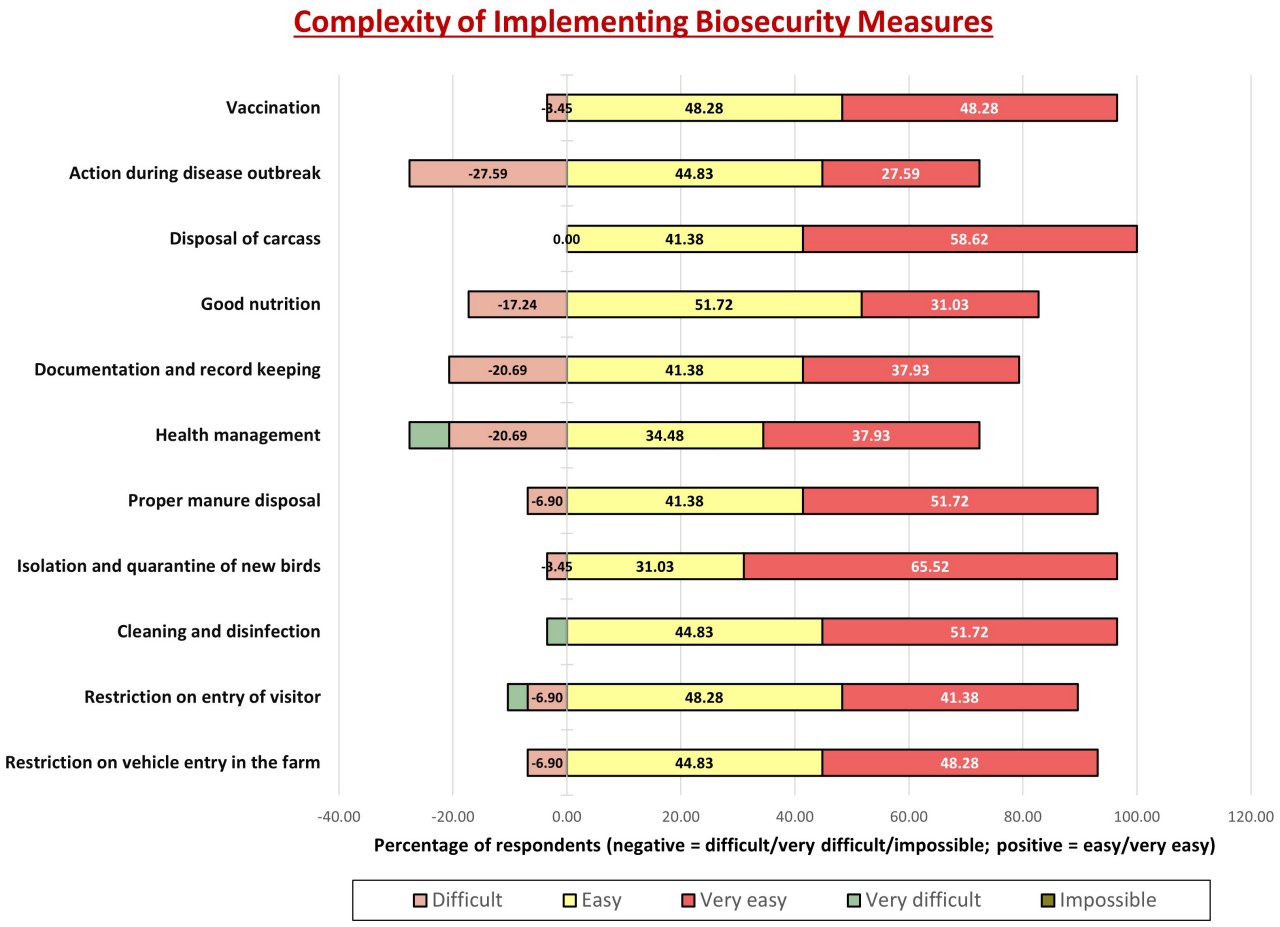
Perceived complexity of implementing biosecurity measures among poultry farmers in Gujarat. Bars represent the proportion of farmers rating each measure as very easy, easy, difficult, very difficult, or impossible to implement. Vaccination, carcass disposal, cleaning and disinfection, and manure disposal were often perceived as difficult or very difficult, while documentation/record keeping and restriction of entry (visitors and vehicles) were also reported as challenging. Isolation and quarantine of new birds was perceived as the most difficult measure. In contrast, practices such as good nutrition and health management were more frequently rated as easy to implement. Together with Figure 5, these findings reveal that although farmers generally recognize the importance of biosecurity measures, practical barriers limit their consistent adoption. The response category “Impossible” was included in the questionnaire but is not represented in the graph, as no participants selected this option.

Graph 5 (Figure 6) revealed that cleaning, vehicle restrictions, and isolation were seen as manageable by over 50%. Disease management and documentation were considered more challenging.

**Figure 7:**
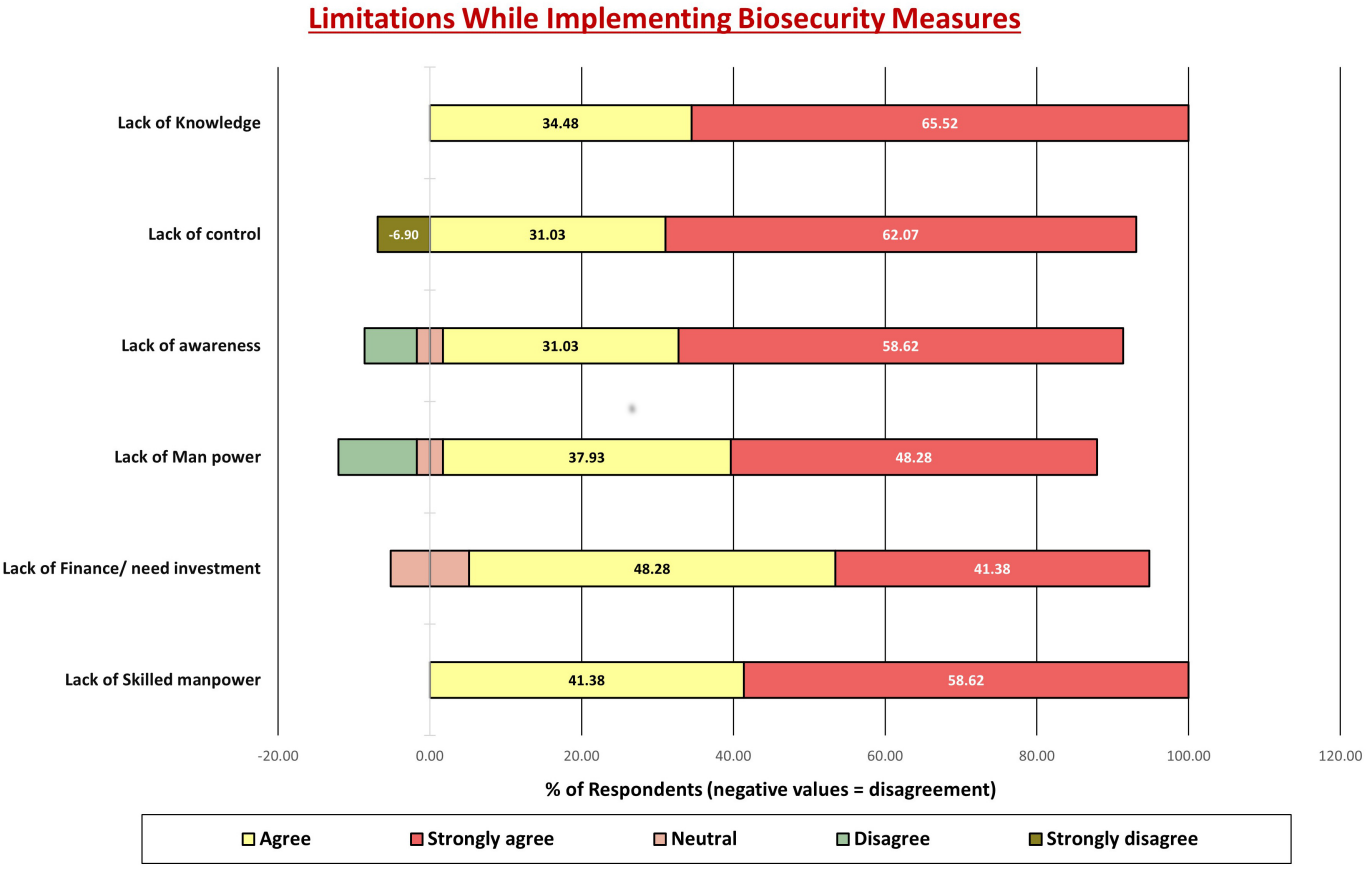
Perceived limitations in implementing biosecurity measures among poultry farmers in Gujarat. Bars represent the proportion of respondents who strongly agreed, agreed, were neutral, disagreed, or strongly disagreed with each limitation. The most frequently reported barriers were lack of knowledge (65.5% strongly agreed), lack of awareness (58.6%), and lack of skilled manpower (58.6%). Financial constraints (48.3% agreement) and manpower shortages (37.9%) were also recognized as limiting factors. Together with Figures 5, 6, these findings highlight that while farmers generally acknowledge the importance of biosecurity and recognize implementation challenges, structural limitations—including training gaps, resource constraints, and inadequate technical support—remain major obstacles to adoption.

Graph 6 (Figure 7) highlighted key barriers: lack of skilled manpower (100%), financial constraints (89.66%), and limited awareness and knowledge (89.65%).

### 3.7 Epidemiological nodes in broiler distribution network

**Figure 8:**
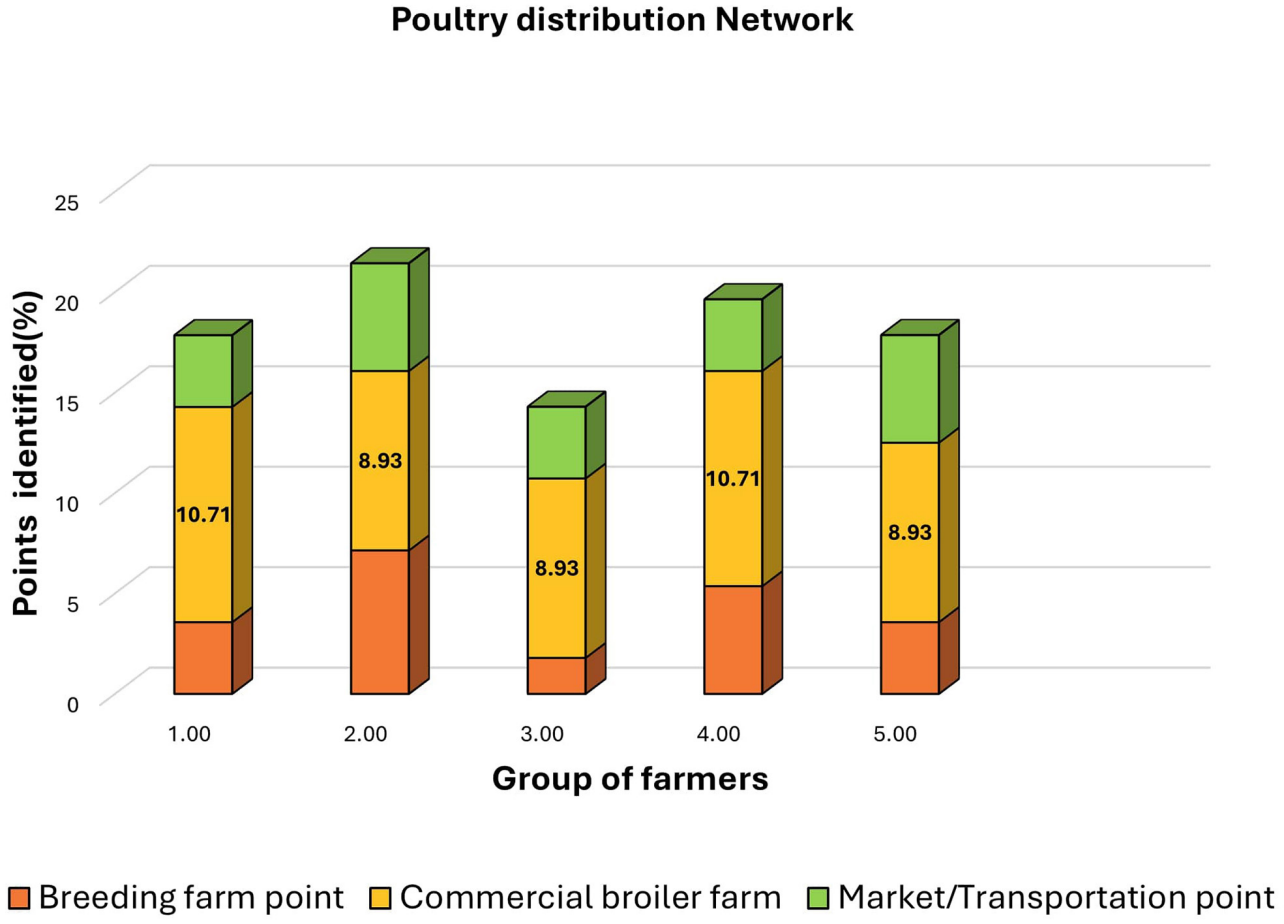
Poultry distribution network showing epidemiological nodes identified by farmer focus groups across breeding farms, commercial broiler farms, and market/transportation points.

In Graph 7 (Figure 8) five focus groups evaluated disease entry risks across breeding farms, broiler farms, and markets. Each group had seven–eight members. Across groups, commercial farms had a consistent 14-point assessment.

- Group 1: identified 6/56 risks (10.71%).
- Group 2: identified 5/56 (8.93%).
- Group 3: identified 5/56 (8.93%).
- Group 4: identified 6/56 (10.71%).
- Group 5: identified 5/56 (8.93%).

Group-wide identification ranged from 14.29 to 21.43%, reflecting key vulnerabilities in disease management across the poultry value chain.

## 4 Discussion

### 4.1 Farmer profile

#### 4.1.1 Marital and family structure

As shown in the results, nearly all surveyed farmers were married (94.87%) and belonged to joint families (94.9%). This demographic pattern reflects the traditional rural social structure of Gujarat, where extended family systems enable labor sharing and collective decision-making in farming activities. The dominance of joint families may facilitate biosecurity implementation through shared responsibility and workforce availability. In contrast, the small proportion of nuclear families (5.1%) may indicate a gradual shift toward individual ownership models, as seen in other modernizing agricultural regions. Previous studies have reported a higher proportion of nuclear families among poultry farmers, suggesting regional variability in household organization but a similar trend in marital stability supporting farm management (13).

#### 4.1.2 Farming experience

Results revealed that 76.92% of farmers had less than 15 years of experience, while only 5.13% had more than 30 years. This indicates a relatively young and emerging farming population, characterized by enthusiasm but limited long-term technical exposure. The findings parallel trends observed in Morocco, where the majority of poultry farmers had under 10 years of experience (14). The prevalence of early-career farmers emphasizes the importance of continuous training programs to strengthen technical skills and enhance awareness of biosecurity and disease management.

#### 4.1.3 Occupational engagement

More than 64% of respondents relied solely on poultry farming, underlining its economic significance as a primary livelihood. Approximately one-third combined poultry with agriculture, demonstrating income diversification strategies that mitigate market or production risks. This aligns with earlier surveys reporting similar patterns of livelihood diversification (15). The negligible proportion of farmers with off-farm employment further underscores dependence on poultry income, which may heighten vulnerability to disease-related losses and reinforce the need for biosecurity investment.

#### 4.1.4 Age distribution

The age structure, with equal representation of farmers aged 20–35 and 35–50 years (44% each), indicates active participation of both young and middle-aged groups in broiler production. Only 13% were older than 50 years, suggesting gradual generational renewal within the sector. These findings are consistent with previous research showing similar age trends (16, 17). The balanced distribution reflects both innovation potential and operational maturity, beneficial for adopting modern biosecurity practices.

#### 4.1.5 Educational background

All respondents possessed formal education—an encouraging sign of informed decision-making capacity. Over half (51%) completed secondary education, and nearly one-third were graduates, indicating a well-educated farming base. Such education levels correlate positively with technology adoption and farm record-keeping (18). These findings suggest that educational attainment can serve as an enabling factor for effective biosecurity training and evidence-based farm management (19).

### 4.2 Farm profile

#### 4.2.1 Farm size and flock distribution

The results showed wide variation in flock size (0–130,000 birds; mean = 20,579), with medium-scale operations dominating (46%). This distribution aligns with previous studies that found similar production ranges economically sustainable for independent farmers (20). Large-scale farms (>10,000 birds) likely leverage economies of scale and mechanization, whereas small-scale farms (< 5,000 birds) rely on localized markets with lower investment but reduced biosecurity infrastructure.

#### 4.2.2 Ownership and contract farming

Independent ownership (69.2%) was more common than contract farming (30.8%), suggesting a strong preference for autonomy. However, contract arrangements provided technical and financial backing to less-experienced farmers. Similar proportions were reported in other regions (21) reinforcing that ownership model choice directly influences access to resources and adherence to standardized biosecurity protocols.

#### 4.2.3 Financing and investment sources

Most farmers (74.4%) relied on personal funds, while only 12.8% accessed bank credit. This cautious financing pattern limits large-scale upgrades in infrastructure and biosecurity measures. Earlier studies in Bangladesh also highlighted reliance on self-financing, indicating a broader trend of limited institutional financial support in poultry farming sectors of South Asia.

#### 4.2.4 Production cycles and management

Farms averaged 5.13 production cycles per year, demonstrating operational regularity comparable to other commercial broiler systems (22). This consistency supports continuous production but may restrict downtime for thorough cleaning and disinfection between cycles, thereby elevating biosecurity risk if not properly managed.

#### 4.2.5 Infrastructure, operational capacity, and biosecurity risks

There's wide variability in shed capacity and number of sheds. Larger farms may have better mechanization, while smaller farms face limitations in infrastructure and funding (23). Most farms are owner-managed with limited personnel, potentially restricting innovation and biosecurity implementation (24). Although 92% of farms had storage facilities, only 72% were rodent-proof, and more than half lacked vehicle-sanitization points—a major biosecurity gap (25). Limited control of human movement also increases the risk of pathogen introduction. Fencing and waste-disposal systems were widely adopted, and farms located away from water bodies reduced wild-bird exposure, though surrounding trees occasionally attracted wild species (26). Consistent farm layout, staff training, and routine sanitation remain vital for sustained biosecurity (27).

### 4.3 Biosecurity profile

The study demonstrated variability in biosecurity awareness and practice, revealing strengths in cleaning, vaccination, and health management but weaknesses in record-keeping, vehicle control, and antimicrobial resistance (AMR) awareness.

#### 4.3.1 Knowledge and awareness of biosecurity and AMR

Despite universal acknowledgment of biosecurity's importance, only 15% of respondents reported good to excellent understanding, and 79.49% were unaware of AMR. These results mirror observations from small-scale poultry systems in other developing countries (28). Limited formal training (61.54% untrained) represents a missed opportunity for systematic capacity building. Enhancing structured farmer education can bridge this gap and promote prudent antimicrobial use (29).

#### 4.3.2 Cleaning and disinfection

All farmers practiced routine cleaning and disinfection, reflecting strong baseline compliance. The majority also used water sanitizers and observed downtime between flocks, reinforcing pathogen-control awareness. However, standardization of sanitizer concentration and cleaning frequency should be promoted through training to ensure consistency.

#### 4.3.3 Isolation and quarantine

High adoption of isolation and quarantine for sick (100%) and new birds (87%) indicates solid understanding of core disease-prevention principles. Nevertheless, lax vehicle restrictions (69.23% unrestricted) and inconsistent visitor control (46.15% unrestricted) remain major weaknesses (30, 31). These findings suggest that while conceptual awareness exists, practical enforcement requires better logistical and infrastructural support.

#### 4.3.4 Restricted access

Access management across farms was inconsistent—only 31% restricted vehicle entry and 54% restricted visitors. Yet, nearly all prevented wild bird access, showing selective implementation. The imbalance reflects prioritization of visible risks (wildlife) over routine vectors like vehicles and personnel (24, 30). Standardized visitor logs, disinfection bays, and physical barriers could close these loopholes.

#### 4.3.5 Dead bird, waste and manure management

Burial was the primary carcass-disposal method (97%), confirming economic practicality but raising environmental concerns when compared to incineration (32, 33). Improper manure storage in open areas and limited use of sealed tanks underline infrastructural and cost barriers (34). Policies promoting safe disposal technologies and integrated waste management could mitigate contamination and vector attraction.

#### 4.3.6 Health management

The universal adoption of vaccination and strong reliance on veterinarians demonstrate commendable preventive measures (35). Nonetheless, high prevalence of diseases such as Colisepticaemia (92.3%) and Avian Influenza (64.1%) signals persistent biosecurity lapses and environmental exposure (36, 37). Strengthening documentation, monitoring, and inclusion of traditional ethnoveterinary alternatives could diversify health strategies and improve resilience.

### 4.4 Factorial analysis of mixed data

The Factorial Analysis of Mixed Data (FAMD) identified farm size, labor availability, and several biosecurity parameters—such as record-keeping, downtime duration, and disease response—as the most influential variables explaining inter-farm variation. Together, Dimensions 1 and 2 accounted for 24.57% of the total variance, reflecting moderate structural and behavioral heterogeneity across surveyed farms.

The analysis suggests that larger farms, typically supported by greater labor capacity and better infrastructure, tend to demonstrate improved compliance with record-keeping and sanitation protocols (38, 39). Conversely, smaller farms—while more flexible in operations—struggle to maintain standardized documentation and downtime, primarily due to resource and time constraints. These patterns corroborate previous findings that link biosecurity compliance to farm scale and technical capability (40, 41).

Notably, the positioning of variables such as “disease response” and “record maintenance” far from the FAMD origin indicates their strong discriminatory power in differentiating farm clusters. This underscores the need for targeted interventions addressing procedural and behavioral gaps—especially documentation and outbreak management. Strengthening these aspects would not only enhance disease surveillance but also improve traceability during outbreaks, a cornerstone of effective One Health implementation.

### 4.5 Hierarchical cluster analysis (HCA)

The Hierarchical Cluster Analysis (HCA) provided complementary insights by grouping farms into three distinct clusters, each characterized by different management practices, educational levels, and biosecurity compliance. This classification enables a more nuanced understanding of the farm typologies existing within Gujarat's broiler sector.

Cluster 1—comprising 74.35% of farms—was typified by good infrastructure but poor practical biosecurity adherence. The paradox of high education levels (all postgraduates) yet poor practices such as keeping dead birds inside sheds or lacking footbaths highlights a knowledge–practice gap. This reflects the broader challenge in agricultural extension, where theoretical awareness often fails to translate into consistent on-ground behavior. Tailored refresher trainings emphasizing practical demonstrations could help bridge this gap.

Cluster 2 (12.83%) represented the most biosecure group, exhibiting proper manure disposal, effective footbath usage, and strong management oversight. Interestingly, none of these respondents had postgraduate qualifications, supporting the idea that applied training and experiential learning may outweigh formal education in promoting behavioral change.

Cluster 3 (12.83%) corresponded to contract-based operations characterized by limited autonomy and infrastructure. The absence of storage rooms and full dependency on integrators likely restrict long-term investment in biosecurity infrastructure. Policies encouraging shared responsibility between contracting companies and growers—such as cost-sharing for disinfection facilities—could enhance compliance in such systems.

Overall, these clusters highlight the existence of diverse operational realities in the same geographic region, echoing similar HCA-based studies in European poultry sectors (42). Recognizing these differences allows policymakers and veterinarians to design differentiated, context-appropriate biosecurity strategies instead of one-size-fits-all guidelines.

### 4.6 Post training evaluation

The post-training evaluation offered an important measure of the intervention's effectiveness in shifting farmers' perceptions and intended behaviors. As reflected in Figures 5–7, there was a clear improvement in understanding and appreciation of core biosecurity principles following the training. More than 70% of farmers agreed on the importance of manure disposal, proper nutrition, and proactive health management, suggesting enhanced conceptual awareness. Similarly, responses for isolation, quarantine, and vaccination practices indicated a positive attitude toward disease prevention, aligning with previous training-based impact assessments (32, 39, 43).

However, a subset of practices—such as vehicle entry control, carcass disposal, and wild bird exclusion—elicited divided opinions, suggesting that practical limitations (infrastructure, cost, and manpower) continue to constrain adoption. This divergence reveals that awareness alone does not ensure compliance; it must be supported by enabling resources and sustained monitoring.

#### 4.6.1 Implementation challenges

While 45%−52% of respondents found routine measures like cleaning, visitor control, and footbath maintenance easy to implement, more technical interventions (such as record-keeping, vaccination logistics, and outbreak management) were perceived as moderately to very difficult (21%−48%). These findings underline the importance of continued on-site mentoring beyond one-time training sessions. Establishing demonstration farms and regular follow-up visits could help reinforce behavioral consistency (44).

#### 4.6.2 Key barriers to biosecurity implementation

The most prominent barriers—shortage of skilled labor (100%), financial limitations (89.66%), and low awareness (89.65%)—mirror structural constraints commonly reported in developing livestock sectors (39, 45). Addressing these challenges requires multi-level collaboration. Financial support through government or cooperative schemes can help farmers invest in infrastructure, while extension services can ensure localized problem-solving. Integrating biosecurity modules into state-level poultry development programs would institutionalize training impact.

Ultimately, the post-training evaluation highlights that knowledge transfer, while crucial, must be coupled with access to financial and technical resources to achieve sustainable behavioral change. Continued engagement of veterinary officers and inclusion of women and youth in future training cohorts could further strengthen long-term adoption and resilience in Gujarat's poultry sector.

### 4.7 Epidemiological nodes in the broiler distribution network

The participatory group discussions provided a structured evaluation of risk points across the commercial broiler production and distribution network, identifying critical nodes where disease introduction and transmission are most likely to occur. By engaging farmers, veterinarians, and local stakeholders, the assessment offered a ground-level perspective on vulnerabilities that quantitative models often overlook. The use of participatory epidemiology aligns with international recommendations for context-driven risk assessment in developing livestock systems (46).

#### 4.7.1 Risk identification in the commercial broiler sector

All five focus groups evaluated risk factors related to disease entry and transmission within breeding farms, broiler production units, and live-bird markets. Remarkably, each group achieved a similar total risk score (14 points) for commercial farms, reflecting consistency in recognizing key vulnerabilities such as inadequate vehicle disinfection, visitor movement, and improper carcass handling.

Groups 1 and 4 identified six specific risks (10.71%), while the remaining groups identified five each (8.93%). The uniform scoring pattern suggests that participants possessed a comparable understanding of farm-level disease risks—possibly due to shared exposure to recent awareness programs and veterinary guidance. However, the slight variations between groups indicate differences in field experience and local disease pressures.

The observed consistency across all five groups highlights that the commercial broiler sector, despite its variable scales of operation, faces similar systemic weaknesses—chiefly related to movement control, sanitation, and disposal management. This reinforces the findings from earlier biosecurity assessments that pinpointed these areas as recurrent gaps in disease prevention (47).

#### 4.7.2 Group-level comparison and interpretation

When data from all production sectors were analyzed collectively, performance differences among focus groups became evident.

- Group 2 achieved the highest identification rate (21.43%; 12 risks), followed closely by Group 4 (19.64%; 11 risks).
- Groups 1 and 5 each identified 10 risks (17.86%), while Group 3 recorded the lowest detection rate (14.29%; eight risks).

These variations may be attributed to differences in group composition—particularly the presence of individuals with veterinary backgrounds or previous disease-management experience. Groups demonstrating higher identification capacity appeared to approach the exercise more systematically, assessing risk points across the entire farm-to-market continuum rather than focusing solely on on-farm practices.

This finding mirrors previous participatory epidemiology research emphasizing the value of multidisciplinary engagement for comprehensive risk mapping. The inclusion of both technical experts and field-experienced farmers enhances the accuracy of hazard identification and encourages practical, community-owned mitigation strategies (48).

#### 4.7.3 Implications for disease prevention and One Health

The identification of disease-entry points across breeding farms, broiler farms, and markets underscores the interconnected nature of the poultry value chain. Pathogen transfer through contaminated vehicles, feed, and live-bird trade remains a significant threat, particularly in regions where informal market networks predominate. As demonstrated in this study, even minor lapses—such as shared transport vehicles or the absence of quarantine for new batches—can compromise entire production clusters.

A One Health–aligned intervention strategy should therefore emphasize surveillance integration between farms, transporters, and marketplaces. Establishing traceability systems and enforcing disinfection checkpoints at transfer nodes would substantially reduce cross-farm contamination risks. Furthermore, participatory risk assessments like the one conducted here can be institutionalized as periodic community exercises to sustain awareness and promote adaptive responses to emerging threats (49).

## 5 Conclusion

This study highlights an urgent need to strengthen biosecurity implementation in commercial broiler farms across Gujarat. While fundamental practices—such as cleaning, vaccination, and fencing—are already in place, disparities in knowledge, resource availability, and adherence remain major constraints. Variations observed through FAMD and cluster analysis further emphasize that biosecurity compliance is strongly influenced by farm scale, managerial capacity, and access to technical support. The participatory risk assessment revealed that despite awareness of disease threats, practical gaps in movement control, waste disposal, and record-keeping persist across the production network. Strengthening farmer education, improving veterinary support systems, and promoting the responsible use of antimicrobials are therefore critical to enhancing farm-level disease prevention and resilience. Addressing these elements through a One Health framework will help mitigate the risk of infectious disease transmission and ensure the long-term sustainability of Gujarat's poultry sector.

## Data Availability

The raw data supporting the conclusions of this article will be made available by the authors, without undue reservation.

## References

[1] Ravindran V. Assessment of biosecurity measures against Newcastle disease in commercial poultry farms in Benue state, Nigeria. Sokoto J Vet Sci. (2017) 15:32. doi: 10.4314/sokjvs.v15i3.5

[2] GOI. 20th Livestock Census, 2019. Ministry of Fisheries; Animal Husbandry and Dairying; Government of India [Internet] (2019). Available online at: https://dahd.gov.in/schemes/programmes/animal-husbandry-statistics (Cited Jul 23, 2025).

[3] Open Government Data (OGD) Platform India. (2022). Available online at: https://data.gov.in (Accessed December 2, 2025).

[4] Samuel Olugbenga O Olusegun Abayomi O Adebimpe Oluseye A Akinbowale Taiwo T. Optimized nutrients diet formulation of broiler poultry rations in Nigeria using linear programming. J Nutr Food Sci S. (2015) 14:002. doi: 10.4172/2155-9600.S14-002

[5] Van Boeckel TP Pires J Silvester R Zhao C Song J Criscuolo NG . Global trends in antimicrobial resistance in animals in low- and middle-income countries. Science. (2019) 365:eaaw1944. doi: 10.1126/science.aaw1944 (Accessed November 8, 2025). 31604207

[6] FAO Biosecurity Toolkit. Available online at: https://www.fao.org/4/a1140e/a1140e00.htm (Accessed July 23, 2025).

[7] King LC Anderson TK Behravesh CB Binkley LE. Zoonotic Diseases in Animal Agriculture and Beyond: A One Health Perspective. Council for Agricultural Science and Technology (2022). doi: 10.5555/20220573915

[8] Gholamian B Shahnazi H Hassanzadeh A. The effect of educational intervention based on BASNEF model for reducing internet addiction among female students: a quasi-experimental study. Ital J Pediatr. (2019) 45:164. doi: 10.1186/s13052-019-0761-4 31856869 PMC6921507

[9] Humphery TJ Henley A Lanning DG. The colonization of broiler chickens with *Campylobacter jejuni*: some epidemiological investigations. Epidemiol Infect. (1993) 110:601–7. doi: 10.1017/S0950268800051025 8519325 PMC2272299

[10] Tablante NL Myint MS Johnson YJ Rhodes K Colby M Hohenhaus G . survey of biosecurity practices as risk factors affecting broiler performance on the Delmarva Peninsula. Avian Dis. (2002) 46:730–4. doi: 10.1637/0005-2086 12243542

[11] Chauvin C Bouvarel I Beløeil P-A Orand J-P Guillemot D Sanders P . pharmaco-epidemiological analysis of factors associated with antimicrobial consumption level in turkey broiler flocks. Vet Res. (2005) 36:199–211. doi: 10.1051/vetres:2004064 15720973

[12] Poultry Disease Prevention Checklist. The Poultry Site. Available online at: https://www.thepoultrysite.com/articles/poultry-disease-prevention-checklist (Accessed July 23, 2025).

[13] Bikash Borthakur BB Pulin Hazarika PH Saharia KK. Socioeconomic and Psychological Status of Poultry Farmers in Dibrugarh District of Assam. (2010). Available online at: https://www.cabidigitallibrary.org/doi/full/10.5555/20103173340 (Accessed November 8, 2025).

[14] Fagrach A Fellahi S Challioui MK Arbani O Zirani IE Kichou F . Backyard poultry flocks in Morocco: demographic characteristics, husbandry practices, and disease and biosecurity management. Anim Open Access J MDPI. (2023) 13:202. doi: 10.3390/ANI13020202 36670742 PMC9854736

[15] Surendra Chaudhary D Sharma S Faran NK Nagar MK. A survey of poultry farmers of Udaipur district regarding the farm demographics, production, hygiene and biosecurity measures. Int J Vet Sci Anim Husb. (2024) 9:391–4.

[16] Kouam MK Jacouba M Nsangou IN Teguia A. Assessment of biosecurity level in small-scale broiler farms in the Western highlands of Cameroon (Central Africa). Trop Anim Health Prod. (2018) 50:1529–38. doi: 10.1007/s11250-018-1591-x 29687218

[17] Billah SM Nargis F Hossain ME Howlider MAR Lee SH. Family poultry production and consumption patterns in selected households of Bangladesh. J Agric Ext Rural Dev. (2013) 5:62–59. doi: 10.5897/JAERD12.113

[18] Tsegaye D Tamir B Gebru G. Assessment of biosecurity practices and its status in small- and medium-scale commercial poultry farms in Arsi and East Showa Zones, Oromia, Ethiopia. Poultry. (2023) 2:334–8. doi: 10.3390/poultry2020025

[19] Niemi JK Sahlström L Kyyrö J Lyytikäinen T Sinisalo A. Farm characteristics and perceptions regarding costs contribute to the adoption of biosecurity in Finnish pig and cattle farms. Rev Agric Food Environ Stud. (2016) 97:215–23. doi: 10.1007/s41130-016-0022-5

[20] Sailo L. A. study on socio-economic profile of the broiler farmers in Mizoram. Int J Bio-resour Stress Manag. (2018) 9:1876. doi: 10.23910/IJBSM/2018.9.3.1876

[21] Baliyan S. Socio-economic factors as determinants of farm management skills among broiler poultry producers in Botswana. Int J Agric Econ. (2017) 2:27. doi: 10.11648/j.ijae.20170202.11

[22] Duarte da Silva Lima N de Alencar Nääs I Garcia RG Jorge de Moura D. Environmental impact of Brazilian broiler production process: evaluation using life cycle assessment. J Clean Prod. (2019) 237:117752. doi: 10.1016/J.JCLEPRO.2019.117752

[23] Mahanty S Doron A Hamilton R. A policy and research agenda for Asia's poultry industry. Asia Pac Policy Stud. (2023) 10:63–72. doi: 10.1002/app5.377

[24] Panda P Tiwari R Singh A Kumari M Singh K Dutt T. Awareness and adoption of farm biosecurity practices in commercial dairy, pig and poultry farms of Uttar Pradesh (India). Trop Anim Health Prod. (2024). 56:203. https://link.springer.com/article/10.1007/s11250-024-04054-3 (Accessed November 12, 2025). 10.1007/s11250-024-04054-3 38995510

[25] Biosecurity Risks to Poultry Farms. Business Queensland. Available online at: https://www.business.qld.gov.au/industries/farms-fishing-forestry/agriculture/animal/industries/poultry/biosecurity/risks (Accessed July 23, 2025).

[26] Biosecurity at the Poultry Farm: A Basic Tool to Ensure Poultry Health and Welfare. Laying Hens. Available online at: https://layinghens.hendrix-genetics.com/en/articles/biosecurity-at-the-poultry-farm-a-basic-tool-to-ensure-poultry-health-and-welfare/ (Accessed July 23, 2025).

[27] Defend the Flock. Available online at: https://www.aphis.usda.gov/livestock-poultry-disease/avian/defend-the-flock (Accessed July 23, 2025).

[28] Rousham EK Unicomb L Islam MA. Human, animal and environmental contributors to antibiotic resistance in low-resource settings: integrating behavioural, epidemiological and One Health approaches. Proc Biol Sci. (2018) 285:20180332. doi: 10.1098/rspb.2018.0332 29643217 PMC5904322

[29] Greru C Thompson R Gowthaman V Shanmugasundaram S Ganesan N Gopala Krishna Murthy TR . A visualisation tool to understand disease prevention and control practices of stakeholders working along the poultry supply chain in southern India. Humanit Soc Sci Commun. (2022) 9:169. doi: 10.1057/s41599-022-01188-3

[30] Samanta I Joardar SN Das PK. Biosecurity strategies for backyard poultry: a controlled way for safe food production. In:Maria Holban A Mihai Grumezescu A, editors. Food Control and Biosecurity. Amsterdam: Elsevier (2018). p. 481–517. doi: 10.1016/B978-0-12-811445-2.00014-3

[31] Kolluri G Tyagi JS Sasidhar PVK. Research Note: Indian poultry industry vis-à-vis coronavirus disease 2019: a situation analysis report. Poult Sci. (2021) 100:100828. doi: 10.1016/J.PSJ.2020.11.011 33516485 PMC7674013

[32] Biosecurity Measures for Profitable Poultry Production in India. SR Publications. Available online at: https://www.srpublication.com/biosecurity-measures-for-profitable-poultry-production-in-india/ (Accessed July 23, 2025).

[33] Zhang L Ren J Bai W. A review of poultry waste-to-wealth: technological progress, modeling and simulation studies, and economic- environmental and social sustainability. Sustainability. (2023) 15:5620. doi: 10.3390/SU15075620

[34] Symeon GK Akamati K Dotas V Karatosidi D Bizelis I Laliotis GP. Manure management as a potential mitigation tool to eliminate greenhouse gas emissions in livestock systems. Sustainability. (2025) 17:586. doi: 10.3390/SU17020586

[35] Thornton PK. Livestock production: recent trends, future prospects. Philos Trans R Soc B Biol Sci. (2010) 365:2853–67. doi: 10.1098/RSTB.2010.0134 20713389 PMC2935116

[36] OIE. Terrestrial Animal Health Code. (2019). Available online at: https://scholar.google.com/scholar?q=OIE.+(2019).+Terrestrial+animal+health+code.+World+Organisation+for+Animal+Health.&hl=en&as_sdt=0,5 (Accessed July 23, 2025).

[37] Global Action Plan on Antimicrobial Resistance. Available online at: https://iris.who.int/handle/10665/193736 (Accessed July 23, 2025).

[38] Pagès J. Analyse factorielle de données mixtes. Rev Stat Appl. (2004) 52:93–111.

[39] Conan A Goutard FL Sorn S Vong S. Biosecurity measures for backyard poultry in developing countries: a systematic review. BMC Vet Res. (2012) 8:240. doi: 10.1186/1746-6148-8-240 23216706 PMC3538710

[40] Tham-Agyekum EK. Assessing farm record keeping behaviour among small-scale poultry farmers in the Ga East Municipality. J Agric Sci. (2010) 2:52. doi: 10.5539/jas.v2n4p52

[41] Delabouglise A Thanh NTL Xuyen HTA Nguyen-Van-Yen B Tuyet PN Lam HM . Poultry farmer response to disease outbreaks in smallholder farming systems in southern Vietnam. Elife. (2020) 9:e59212. doi: 10.7554/eLife.59212 32840482 PMC7505654

[42] Delpont M Guinat C Guérin JL Vaillancourt JP Paul MC. Biosecurity measures in French poultry farms are associated with farm type and location. Prev Vet Med. (2021) 195:105466. doi: 10.1016/j.prevetmed.2021.105466 34419776

[43] Tilli G Laconi A Galuppo F Mughini-Gras L Piccirillo A. Assessing biosecurity compliance in poultry farms: a survey in a densely populated poultry area in North East Italy. Animals. (2022) 12:1409. doi: 10.3390/ani12111409 35681871 PMC9179503

[44] Delpont M Salazar LG Dewulf J Zbikowski A Szeleszczuk P Dufay-Lefort AC . Monitoring biosecurity in poultry production: an overview of databases reporting compliance with biosecurity practices from 7 European countries. Front Vet Sci. (2023) 10:1231377. doi: 10.3389/fvets.2023.1231377 37649565 PMC10465163

[45] Asfaw YT Ameni G Medhin G Gumi B Wieland B. Poultry health services in Ethiopia: availability of diagnostic, clinical, and vaccination services. Poult Sci. (2021) 100:101023. doi: 10.1016/J.PSJ.2021.101023 33872862 PMC8080080

[46] Chantziaras I Boyen F Callens B Dewulf J. Correlation between veterinary antimicrobial use and antimicrobial resistance in food-producing animals: a report on seven countries. J Antimicrob Chemother. (2014) 69:827–34. doi: 10.1093/jac/dkt443 24216767

[47] Lockhart CY Stevenson MA Rawdon TG Gerber N French NP. Patterns of contact within the New Zealand poultry industry. Prev Vet Med. (2010) 95:258–66. doi: 10.1016/J.PREVETMED.2010.04.009 20569999

[48] Guinat C Artois J Bronner A Guérin JL Gilbert M Paul MC. Duck production systems and highly pathogenic avian influenza H5N8 in France, 2016–2017. Sci Rep. (2019) 9:6177. doi: 10.1038/s41598-019-42607-x 30992486 PMC6467959

[49] Jand SK Kaur P Sharma NS. Mycoses and mycotoxicosis in poultry: a review. Indian J Anim Sci. (2005) 75.

