# Supplementary material for: Evaluating biosecurity implementation in commercial broiler poultry production in Gujarat
Source: Front Vet Sci. 2026 Jan 28;12:1699509. doi: 10.3389/fvets.2025.1699509 (PMC12892967; doi:10.3389/fvets.2025.1699509)
Supplement: Supplementary file 1 [file Table_1.docx]

**Supplementary Table S1. Criteria included in the farmer, farm, and biosecurity profiles.**

This table provides the detailed criteria covered in the survey questionnaire, including farmer demographics, farm characteristics, and adoption of biosecurity measures. Information spans knowledge and familiarity with biosecurity and antimicrobial resistance (AMR), farm hygiene and management practices, visitor and vehicle access, waste disposal, health management, and preventive measures.

| **Criteria** | **Details** |
| --- | --- |
| Farmer Profile | - Name, age & gender - Education, - Contact detail and address, - Marital status, - Family details, - Land holding, - Approximate annual family income, - Source of income, - Experience in poultry farming |
| Farm Profile | - Farm name, address & registration details, Types of farms & mode of operation, - Number and category of poultry birds kept & production cycle, - Capacity and number of poultry sheds, - Rearing system, - Number of staff |
| Biosecurity Profile | - Familiarity and knowledge about the term biosecurity, - Adoption about the different biosecurity measures at the farm, - Cleaning of water drinker and nipple, - Quality of a day-old chicks, - Storeroom safety, - Removal of dead birds and little material, - Visiting hours of farms and preventive measures followed by visitors, - Details about vehicles’ entry at farm and their disinfectant facility, - Protective measures followed by labors at farm, - Encounter diseases and their symptoms at the farm, - Disease transmission & their control, and Vaccination program, - Awareness about antibiotics and their use, - Familiarity with the term AMR, - Ethno-veterinary medicine practices adopted at farm, - Isolation and disposal of sick and dead birds, - Details about manure collection and its disposal, - Availability of natural water resources around farm area, - Direct contact of wild birds to the farm, - Distance from another nearby farm |
