# Supplementary material for: Evaluating biosecurity implementation in commercial broiler poultry production in Gujarat
Source: Front Vet Sci. 2026 Jan 28;12:1699509. doi: 10.3389/fvets.2025.1699509 (PMC12892967; doi:10.3389/fvets.2025.1699509)
Supplement: Supplementary file 2 [file Table_2.docx]

**Supplementary Table S2. Detailed biosecurity practices among commercial broiler farms in Gujarat.**
This table provides a full parameter-wise breakdown of biosecurity practices, including farmers’ knowledge and awareness, cleaning and disinfection, isolation and quarantine, restricted access, waste and manure management, and health management. Frequencies and percentages for each response are shown (n = 39 farms). A condensed summary is provided in Table 3 of the main manuscript.

| **Biosecurity Profile** | | | | |
| --- | --- | --- | --- | --- |
| **Characteristics** | **Categories** | **Total Observations** | **Parameters** | |
| **Farmers' Knowledge About Biosecurity** | |  | **Frequency** | **Percentage** |
| Scale of biosecurity knowledge | No | 39 | 0 | 0.00% |
|  | Very poor |  | 16 | 41.03% |
|  | Poor |  | 7 | 17.95% |
|  | Good |  | 10 | 25.64% |
|  | Very good |  | 5 | 12.82% |
|  | Excellent |  | 1 | 2.56% |
| Idea about the transmission of diseases by pests /flies/insects | | 39 | 22 | 56% |
| Awareness about AMR | | 39 | 8 | 21% |
| **Cleaning and Disinfection** | | | | |
| Cleaning of shed | | 39 | 39 | 100% |
| Cleaning of surrounding area of shed | | 39 | 39 | 100% |
| Use of water sanitizer | | 39 | 28 | 72% |
| Disinfection of shed | | 39 | 39 | 100% |
| Adoption of rodent control | | 39 | 39 | 100% |
| Adoption of pest control | | 39 | 39 | 100% |
| Ensure shed cleanliness before placing the DOC (Day old chicks) | | 39 | 39 | 100% |
| **Isolation and Quarantine** | | | | |
| Isolation and quarantine of new birds | | 39 | 34 | 87% |
| Minimum required period to keep shed empty between two batches | 0 day | 39 | 1 | 3% |
|  | 10 days |  | 22 | 56% |
|  | 21 days |  | 16 | 41% |
|  | 30 days |  | 0 | 0% |
| Isolation of sick bird | | 39 | 39 | 100% |
| **Restricted Access** | | | | |
| Restriction on vehicle entry in the farm | | 39 | 12 | 31% |
| Record keeping for vehicle entry | |  | 17 | 44% |
| Restriction on entry of visitors | |  | 22 | 56% |
| Preventive follow-up by visitors | |  | 17 | 44% |
| Prevention of contact with wild birds | |  | 23 | 59% |
| **Dead Bird, Waste and Manure Management** | | | | |
| Dead bird disposal method | Burial | 39 | 38 | 97% |
|  | Incineration |  | 1 | 3% |
|  | Chemical decomposition |  | 0 | 0% |
|  | Burial and incineration |  | 0 | 0% |
| Dead birds are kept | Outside the shed | 39 | 37 | 95% |
|  | Inside the shed |  | 2 | 5% |
| Washing of hands after handling the sick and dead birds | | 39 | 39 | 100% |
| Proper manure disposal | | 39 | 37 | 95% |
| Storage of poultry manure | | 39 | 8 | 21% |
| Method of storing manure | Inside the tank | 8 | 1 | 13% |
|  | Open farm area |  | 5 | 63% |
|  | Away from the farm |  | 2 | 25% |
| Vehicle is employed to lift the manure | | 39 | 37 | 95% |
| **Health Management** | | | | |
| Health management | | 39 | 39 | 100% |
| Good nutrition | | 39 | 31 | 79% |
| Action during disease outbreak | | 39 | 28 | 72% |
| Vaccination | | 39 | 39 | 100% |
| Good quality day old chicks | | 39 | 37 | 95% |
| Use of protective wears by workers | | 39 | 39 | 100% |
| Farm previously encountered disease outbreak | | 39 | 39 | 100% |
| Use of antibiotic in farms | | 39 | 39 | 100% |
| Adoptions of EVM | | 39 | 7 | 18% |
| Advise from veterinarian | | 39 | 39 | 100% |
